# A transcriptional network associated with natural variation in *Drosophila *aggressive behavior

**DOI:** 10.1186/gb-2009-10-7-r76

**Published:** 2009-07-16

**Authors:** Alexis C Edwards, Julien F Ayroles, Eric A Stone, Mary Anna Carbone, Richard F Lyman, Trudy FC Mackay

**Affiliations:** 1 Department of Genetics, North Carolina State University, Raleigh, North Carolina 27695, USA; 2 WM Keck Center for Behavioral Biology, North Carolina State University, Raleigh, North Carolina 27695, USA; 3 Department of Statistics, North Carolina State University, Raleigh, North Carolina 27695, USA; 4 Current address: Virginia Institute for Psychiatric and Behavioral Genetics, Virginia Commonwealth University, Department of Psychiatry, Richmond, VA 23298-0126, USA

## Abstract

**Background:**

Aggressive behavior is an important component of fitness in most animals. Aggressive behavior is genetically complex, with natural variation attributable to multiple segregating loci with allelic effects that are sensitive to the physical and social environment. However, we know little about the genes and genetic networks affecting natural variation in aggressive behavior. Populations of *Drosophila melanogaster *harbor quantitative genetic variation in aggressive behavior, providing an excellent model system for dissecting the genetic basis of naturally occurring variation in aggression.

**Results:**

Correlating variation in transcript abundance with variation in complex trait phenotypes is a rapid method for identifying candidate genes. We quantified aggressive behavior in 40 wild-derived inbred lines of *D. melanogaster *and performed a genome-wide association screen for quantitative trait transcripts and single feature polymorphisms affecting aggression. We identified 266 novel candidate genes associated with aggressive behavior, many of which have pleiotropic effects on metabolism, development, and/or other behavioral traits. We performed behavioral tests of mutations in 12 of these candidate genes, and show that nine indeed affected aggressive behavior. We used the genetic correlations among the quantitative trait transcripts to derive a transcriptional genetic network associated with natural variation in aggressive behavior. The network consists of nine modules of correlated transcripts that are enriched for genes affecting common functions, tissue-specific expression patterns, and/or DNA sequence motifs.

**Conclusions:**

Correlations among genetically variable transcripts that are associated with genetic variation in organismal behavior establish a foundation for understanding natural variation for complex behaviors in terms of networks of interacting genes.

## Abstract 2

A genome-wide screen of inbred Drosophila lines together with transcriptional network modeling reveals insights into the genetic bases of heritable aggression.

## Background

Animals display aggressive behaviors in defense of territory, to secure and defend food and mates, and to establish dominance hierarchies. These behaviors are, however, energetically costly and individually risky, suggesting that excessive aggression may be deleterious. In humans, aggression often manifests as violent behavior with attendant costs to society, and is frequently a component of psychiatric disorders, including schizophrenia, conduct disorder, alcoholism, bipolar disorder, and Alzheimer's disease [1-4]. Analysis of mutations and pharmacological treatments have established that aggressive behavior is evolutionarily conserved and is modulated by the neurotransmitters serotonin, dopamine, norepinephrine, γ-aminobutyric acid, histamine and nitric oxide as well as their receptors and transporters and key enzymes in their biosynthetic pathways in mammals [5] and invertebrates [6]. However, these molecules are not the only players. In mice, mutations in *fierce*, which encodes a nuclear receptor [7], *neural cell adhesion molecule *[8], *interleukin-6 *[9] and *Cathepsin E *[10] affect aggressive behavior. In *Drosophila*, aggressive behavior is correlated with levels of β-alanine [11,12], correct expression of sex-specific transcripts of *fruitless *[13,14], biogenic amines [11,15], and expression of neuropeptide F [15].

Levels of aggression vary continuously in natural populations, due to the segregation of alleles at multiple loci with effects that depend on the social and physical environment: aggressive behavior is thus a typical quantitative trait [16]. In contrast to our understanding of the neurobiological and genetic mechanisms responsible for the manifestation of aggressive behavior, we know very little of the genes and genetic networks affecting natural variation in aggression. Hints that the genetic architecture of aggressive behavior may be complex come from studies examining correlated responses of the *Drosophila *transcriptome to artificial selection for aggressive behavior in a laboratory stock [17] and a population recently derived from nature [18]. These studies showed that the expression of 80 [17] to 1,539 transcripts [18] involved in a wide variety of biological processes and molecular functions varied between the selected and control lines. Subsequent analysis of the effects of mutations in genes encoding some of these transcripts showed that *Cyp6a20 *[17] and 15 other novel genes [18] (*muscleblind*, *CG17154*, *CG5966*, *CG30015*, *Darkener of apricot*, *CG14478*, *CG12292*, *tramtrack*, *CG1623*, *CG13512*, *SP71*, *longitudinals lacking*, *scribbler*, *Male-specific RNA 87F*, *kismet*) affect aggressive behavior. However, the genotypes created by artificial selection are different from any naturally segregating genotype, and it is possible that novel combinations of alleles perturb the transcriptome beyond the range of variation that would be found in a population of wild-type alleles. In addition, selection induces linkage disequilibrium between selected and linked loci, raising the possibility that some correlated transcriptional responses to selection are due to linkage drag.

Here, we quantified male aggressive behavior for 40 inbred lines derived from the same population, and performed a genome-wide association scan for quantitative trait transcripts (QTTs) [19] and single feature polymorphisms (SFPs) [20] associated with aggressive behavior in wild-type genotypes. This unbiased genomic approach reveals natural genetic variation that is correlated with aggression at the level of allelic differences and networks of genetically correlated transcripts.

## Results and discussion

### Natural variation in aggressive behavior

**Figure 1:**
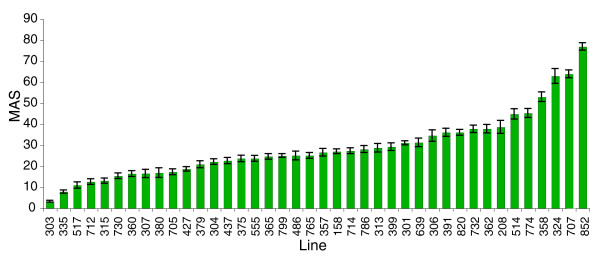
Variation in aggressive behavior among 40 wild-derived inbred lines. The line number is indicated on the x-axis, and the mean aggression score (MAS) on the y-axis. Error bars are standard error.

We quantified aggressive behavior of 40 wild-derived inbred lines, using a rapid and high-throughput behavioral assay [19]. Variation in aggressive behavior was continuously distributed among these lines, as expected for a quantitative trait. There was significant genetic variation in aggression among lines (*F*_40,779 _= 73.0168, *P *< 0.0001; Figure 1). Estimates of among line (*σ*_*L*_^2^) and within line (*σ*_*E*_^2^) variance components were *σ*_*L*_^2 ^= 0.783 and *σ*_*E*_^2 ^= 0.217, for a broad-sense heritability (*H*^2^) of aggressive behavior of *H*^2 ^= 0.78. Surprisingly, there was a 25-fold range of aggressive behavior in these lines: from an average of 3.3 to 76.9 aggressive encounters for 8 flies in a 2-minute observation period.

The variation among the inbred lines far exceeds that of lines selected for 21 generations for increased and decreased aggressive behavior, which only differ less than threefold (with a mean of 14.2 and 34.2 encounters in the high and low selection lines using the same assay) [18]. Under a strictly additive model, we expect variation among fully inbred lines to be twice the additive genetic variation in the base population from which they were derived [16]. Thus, under strict additivity, the estimate of the narrow sense heritability (*h*^2^) in this population would be *h*^2 ^= 0.64. This is much greater than the estimate of realized *h*^2 ^from response to selection (*h*^2 ^≈ 0.09) [18], indicating that alleles affecting natural variation are recessive and/or interact epistatically.

### Candidate genes for aggressive behavior

Previously, we quantified variation in gene expression among these wild-derived inbred lines [21]. A total of 7,508 transcripts were significantly variable among lines in males at a false discovery rate (FDR) of < 0.01 and 3,316 probes contained SFPs. We identified 133 QTTs (*P *< 0.01) associated with variation in aggressive behavior (Additional data file 1). In addition, 167 SFPs (*P *< 0.05) with a minor allele frequency of at least 10% were associated with variation in aggression; these represent 137 independent genes (Additional data file 2). Four of the QTTs were also implicated as candidates from the SFP analysis (*CG1146*, *CG2556*, *CG31038 *and *methuselah-like 8*). No gene ontology information is available for three of these genes (*CG1146*, *CG2556*, and *CG31038*). *methuselah-like 8 *encodes a predicted G protein coupled receptor that may affect the determination of life span [22].

In total, these analyses implicate 266 unique candidate genes associated with natural variation in aggressive behavior. These candidate genes are involved in a broad spectrum of biological processes, including vision, olfaction, learning and memory, and the development and function of the nervous system (Additional data files 1 to 4). However, the candidate genes are also involved in transcription, protein modification, mitosis and other basic cellular processes (Additional data files 1 to 4). More than half of the genes with annotations are involved in metabolism, nearly 60% have protein binding functions, and approximately 25% are implicated in development (Additional data file 5) [23].

Two categories of candidate genes are worthy of mention. We found a member of the Cytochrome P450 gene family associated with aggressive behavior, *Cyp4p2*. Members of this gene family have also been associated with aggressive behavior in previous studies [17,18]. Cytochrome P450s are generally involved in oxidation, metabolism, protection from xenobiotics, and possibly pheromone recognition [24]. The repeated implication of this class of genes suggests that some or all of these functions, or yet unknown functions of this class of proteins, mediate aggressive behavior, although it remains unclear precisely how. We also found three genes that have been previously implicated in learning and/or memory to be associated with natural variation in aggression in this screen - *nord*, *visgun*, and *klingon *[25] - consistent with a previous report that *Drosophila *aggressive behavior is associated with learning and memory [26]. Perhaps variation in these genes affects the fly's learning ability, which could subsequently influence the behavioral response to aggressive encounters. Assessment of these wild-derived lines in a learning and memory assay could inform our understanding of the relevance and variation of social memory in wild *Drosophila*.

A total of 26 of the 266 candidate genes identified in this study overlapped with the candidate genes implicated from the correlated response of the transcriptome to selection for divergent level of aggressive behavior [18], from a different sample of the same base population as the one from which the inbred lines were derived (Additional data file 6). This is no more overlap than expected by chance (χ_1_^2 ^= 0.36, *P *> 0.05). There are several possible - and not mutually exclusive - reasons why the degree of overlap between the two experiments is not more extensive. First, the observation that there is no more overlap between the two experiments than expected by chance could mean that there are many rare alleles affecting aggressive behavior segregating in nature, such that two independent samples captured different subsets of alleles. Second, the flies from the selection lines were not mated, and had been starved for 90 minutes prior to RNA extraction, in contrast to the mated, fully fed flies for which transcript profiles were obtained in this experiment. Third, the control line was the most extreme for many of the transcripts that were divergent among the selection lines; this type of transcript-phenotype association will not be detected in a linear regression. Fourth, selection causes linkage disequilibrium between the selected locus and linked unselected loci; changes in transcript abundance among these linked loci between the selection lines are false positive associations. In contrast, the rapid decay of linkage disequilibrium in regions of normal recombination in unselected *Drosophila *[27,28] minimizes false positive associations of transcript abundance of linked loci in the unselected inbred lines. Fifth, a greater fraction of the genetic variation among the inbred lines than the selection lines is due to dominance and epistasis. The transcriptional signature of a homozygous recessive allele in the inbred lines is likely to be different from the same allele as a heterozygote in the selection lines. Thus, the overlap of genes between the two studies may be enriched for loci with additive effects that causally affect natural variation in aggressive behavior.

### Functional tests

**Figure 2:**
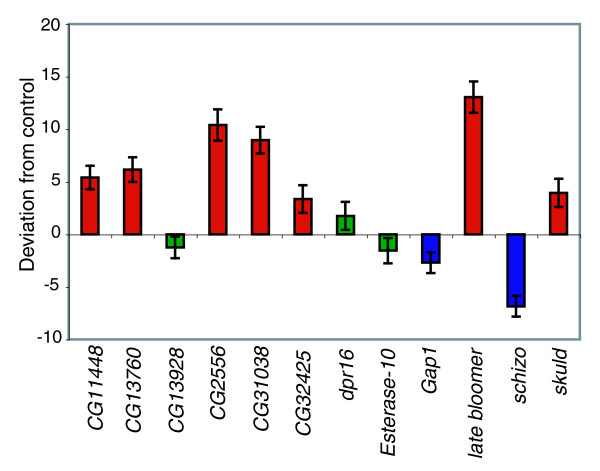
Aggression levels in *P*-element mutants. Mean deviation from control levels of aggression is depicted (± standard error). Red bars indicate significantly higher aggression (*P *< 0.05); blue bars indicate significantly lower aggression; and green bars indicate lines that did not differ significantly from control.

To evaluate whether the candidate genes suggested from these analyses potentially affect aggressive behavior, we assessed aggression levels of *P*-element insertional mutations in 12 of the candidate genes, and their co-isogenic control lines. Nine of the mutant alleles were associated with significantly different aggression levels from the control (Figure 2). This high 'success' rate shows that expression profiling of wild-derived genetically divergent lines is an efficient method for identifying candidate genes affecting complex traits, as has been observed previously [17,18,29-31].

Flies with mutations in *CG11448*, *CG13760*, *CG2556*, *CG31038*, *CG32425*, *late bloomer *and *skuld *are all more aggressive than their controls, while flies with mutations in *GTPase-activating protein 1 *(*Gap1*) and *schizo *are less aggressive than the control strain. No gene ontology information is available for the predicted genes tested; however, *CG11448 *is homologous to the amyloid beta A4 precursor protein, which is implicated in Alzheimer's disease. *late bloomer *has a role in nervous system development and synapse biogenesis. It is homologous to TSPAN7, a tetraspanin protein implicated in mental retardation [32]. *skuld *is involved in numerous transcription-related processes, and also has roles in metabolism and development. *Gap1 *has roles in the cell cycle, and is also involved in signal transduction and numerous developmental processes, such as axis specification and sensory organ development. Finally, *schizo *is involved in several signal transduction pathways, the development of the central nervous system, and muscle development. It is homologous to the human protein ADP-ribosylation factor guanine nucleotide exchange factor 2, dysfunctions of which are associated with microcephaly [33].

### Transcriptional network associated with aggression

**Figure 3:**
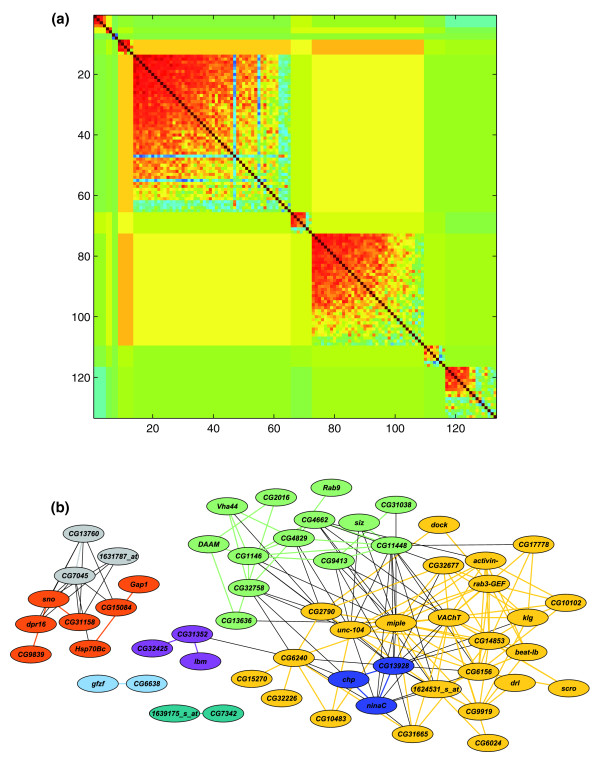
Modules of correlated transcripts associated with variation in aggressive behavior. **(a) **Heat map of correlated probe sets after module formation. The strength of the module decreases down the diagonal. **(b) **Network view of the most highly correlated (*r *≥ 0.7) probe sets where the edges represent correlated transcripts and the color-coding of nodes represents the different modules depicted in (a).

The transcriptome is highly genetically inter-correlated [21]. This correlation structure can be used to infer modules of genetically correlated transcripts associated with aggressive behavior, after removing the correlations among the transcripts attributable to their association with aggression itself. The number and contents of modules are determined such that the average correlation of probe sets within a module is maximized, while the average correlation among probe sets in different modules is minimized. The 133 QTTs grouped into 9 modules, ranging in size from 2 to 54 probe sets (Figure 3a; Additional data file 7). The correlated transcript modules associated with aggressive behavior can also be represented as an interaction network, with edges between transcripts in the network determined by genetic correlations in transcript abundance exceeding a threshold value (Figure 3b represents |*r*| ≥ 0.7). Note that these are, at present, undirected networks. We do not know which transcripts are causally associated with variation in aggression, due to functional polymorphisms in *cis*-regulatory regions, and which transcripts are *trans*-regulated and change expression as a consequence of *cis*-regulatory variation at another locus [34].

**Figure 4:**
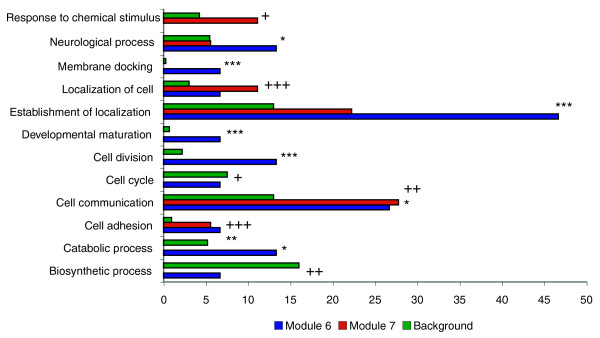
Differences in Gene Ontology representation between modules. All categories depicted are statistically over- or under-represented in module 6 and/or 7 relative to the appropriate genomic background. Asterisks indicate significance levels in module 6, while plus symbols (+) indicate significance in module 7. For example, genes involved in the cell cycle are significantly (*P *< 0.05) under-represented in module 7. */+, *P *< 0.05; **/++, *P *< 0.01; ***/+++, *P *< 0.001.

**Figure 5:**
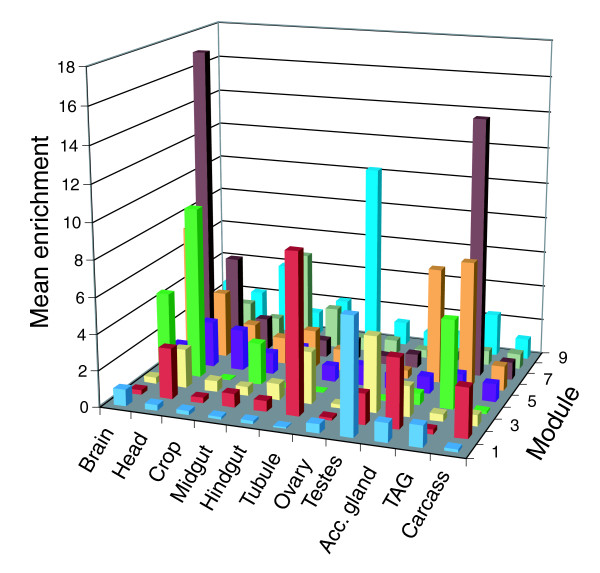
Module-specific enrichment scores in adult tissues, based on data from FlyAtlas [36]. Acc. Gland, accessory gland.

We evaluated the biological plausibility of the modules by querying whether genes in the modules are enriched for shared gene ontology categories, tissue-specific expression patterns, or DNA sequence motifs (the latter using the Multiple EM for Motif Elicitation (MEME) tool). Approximately one-third of the transcripts in module 6 affect ion binding, relative to approximately 2% of the probe sets in the genomic background; this is a significant enrichment (*P *< 0.01). Nearly 50% of the annotated genes in module 6 are involved in establishment of localization, compared to approximately 13% of the background (*P *< 0.001); 25 to 30% of the genes in modules 6 (*P *< 0.05) and 7 (*P *< 0.01) are involved in cell communication, whereas only 13% of the background falls into that category (Figure 4). Module 7 is enriched for several categories related to development (Figure 4). Transcripts in modules 6 and 7 are enriched in the brain, head, and thoracicoabdominal ganglion (Figure 5), indicating that these genes function primarily in central nervous system functions. However, the fact that they fall into distinct modules suggests that their specific functions differ, or that they are differentially regulated in a temporally or spatially specific manner.

**Figure 6:**
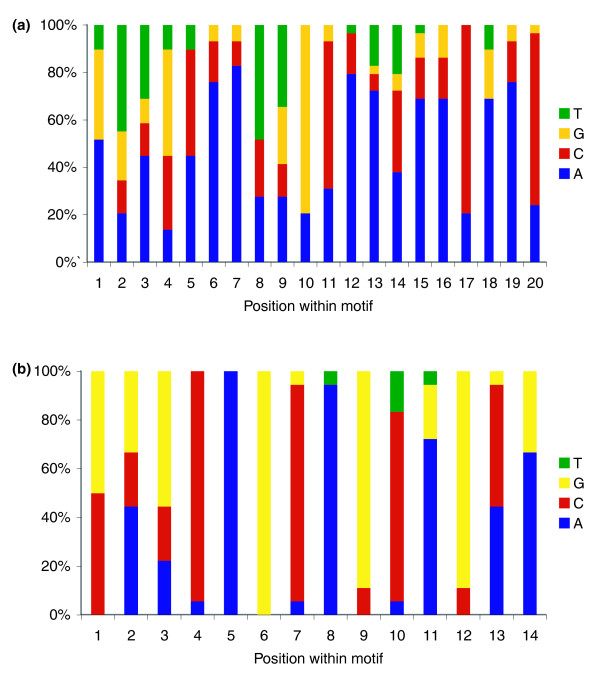
Conserved motifs in modules 6 and 7. **(a,b) **The motifs most frequently found among genes in modules 6 (a) and 7 (b) are shown. The frequency of each nucleotide at each position is depicted on the y-axis, with the nucleotide position within the consensus sequence depicted on the x-axis. The motif in (a) was contained within 29 of 35 genes in module 6; the motif in (b) was contained in 18 of 54 genes in module 7. Significance level of adherence to the consensus sequence was at least *P *= 2.68 × 10^-4 ^for (a) and *P *= 9.32 × 10^-6 ^for (b).

Additional support for the hypothesis that genes in a module are co-regulated is generated by shared MEMEs among members of a module [35] (Figure 6). The *P*-value for each gene containing the consensus sequence represents the probability of a random sequence having the same match score or higher. Of 35 genes in module 6, 29 share a motif with a 20-bp consensus sequence, and the significance values for genes containing this motif range from *P *= 2.68 × 10^-4 ^to *P *= 1.82 × 10^-10 ^(Figure 6a). Of 54 genes in module 7, 18 share a 14-bp motif, with *P*-values ranging from *P *= 9.32 × 10^-6 ^to *P *= 4.73 × 10^-9 ^(Figure 6b).

Although many of the QTTs lack annotation, we can infer potential functions based on the characterized genes that fall into the same correlated module. Three of the four QTTs in module 1 belong to a large transcriptional module enriched for male biased transcripts [21], and these genes are highly expressed in the testis [36]; perhaps this module is related specifically to male reproductive functions. Of the five QTTs in module 4, three are involved in visual perception. Their correlated expression implies that the others, *CG13928 *and *CG6403*, might share a similar function. The fact that all of these transcripts are highly expressed in the head supports this possibility (Figure 5). Three of the four annotated genes in module 8 are involved in metabolic functions, suggesting a similar role for the uncharacterized genes in that module. Additional tests can help us tease apart the relationships among genes within a module. For example, manipulation of a single gene and assessment of the effects on other genes within the same module can elucidate causality and direction of effects.

### Pleiotropy

The wild-derived inbred lines have been assessed for variation in other complex traits: longevity, starvation stress resistance, chill coma recovery time, locomotor reactivity (a startle response), copulation latency, competitive fitness and sleep traits [21,37]. At the level of organismal phenotype, only locomotor reactivity was significantly genetically correlated with aggressive behavior (*r*_*G *_= 0.49, *P *< 0.001). However, organismal genetic correlations can only be significant if alleles affecting both traits have largely similar positive or negative effects on the traits [16]. There can be substantial pleiotropy in the absence of genetic correlation if alleles at many loci affect both traits, but the sign of the effects is not correlated. This motivated us to ask whether particular modules of transcripts associated with aggressive behavior were associated with modules of transcripts associated with the other traits (Additional data file 8). Many of the probe sets implicated in multiple traits correspond to predicted genes about which little is known. However, transcript abundance of *synaptogyrin*, which is involved in synaptic vesicle exocytosis [38], is associated with variation in starvation resistance and fitness [21]. *Rab9 *is associated with chill coma recovery [21] and sleep [37]. *GRHRII*, which encodes a predicted G-protein coupled receptor [39] and gonadotropin-releasing hormone receptor [40], is associated with starvation resistance [21] and sleep [37].

In addition to examining genetic correlations between QTTs affecting aggressive behavior, we can ask which of the genes affecting aggressive behavior are most highly correlated (*r *≥ 0.70) to the transcriptome (Figure 3b). Three QTTs stand out as being highly connected. *miple *transcript abundance is highly correlated with 22 other transcripts. It is highly pleiotropic, and is thought to affect locomotor behavior, muscle development, ATP binding, synapse biogenesis, and response to stimulus [22]. *VAChT *expression is correlated with 21 other transcripts. It is described as an acetylcholine transporter, and is also involved in the response to a chemical stimulus [22]. Another 'hub' gene is *unc-104*, which falls into many of the Gene Ontology categories described for *miple*; it is also involved in nucleotide binding. Mutations in human homologues have been implicated in spastic paraplegia and Charcot-Marie-Tooth disease [22]. Additional highly connected genes are the computationally predicted genes *CG2790*, *CG13928*, *CG14853*, and *CG6156*, about which little annotation information is available, although *CG2790 *and *CG13928 *are reportedly involved in zinc ion and protein binding.

Expression of all of these integral genes is highly enriched in the brain, head, and thoracicoabdominal ganglion. Furthermore, the male accessory glands exhibit enrichment of *unc-104*, and *CG6156 *is up-regulated in the crop, tubule, larval tubule, and larval fat body [36]. Their high degree of connectivity implies that these genes might be central to networks involved in aggressive behavior. The range of biological processes and molecular functions in which they are involved makes it difficult to isolate which are relevant to aggression, but their high expression levels in the head and nervous system unsurprisingly implicate those tissues in the modulation of aggression. We can also use these data to develop hypotheses about the highly connected yet uncharacterized genes *CG2790*, *CG13928*, *CG14853*, and *CG6156*.

### Insights about the genetic architecture of aggressive behavior

Aggression is clearly a highly complex trait - we have identified 266 candidate genes associated with natural variation in aggressive behavior, none of which have been previously implicated to affect aggression. Follow-up functional validation shows that 75% of *P*-element insertional mutations tested in these candidate genes indeed affect aggression. The candidate genes embrace a wide range of biological functions with plausible connections to aggressive behavior (sensory perception and chemosensation, function and development of the nervous system), as well as other general functions with less obvious relationships to aggression *per se *(metabolism, protein modification, mitosis). Analysis of natural variants affecting complex traits that have survived the sieve of natural selection thus gives insights about the genetic basis of complex behaviors that are not possible from analysis of mutations of large effect. That none of the genes previously implicated to affect aggression was detected in this screen is somewhat surprising. There are several possible explanations. The known candidate genes may not be genetically variable at the level of transcription; we could not detect genetically variable transcripts at these loci because they are expressed at low levels or at a different developmental stage; our SFP map detects only a small fraction of polymorphic variants; and the candidate genes may not tolerate functional variation due to strong purifying selection. For example, variation in *fruitless *was not associated with variation in aggressive behavior in this study or previous studies [17,18]. Only one of the seven probe sets on the array that target *fruitless *was genetically variable, and variation in *fruitless *expression for this probe set was not associated with variation in aggressive behavior.

The QTTs associated with natural variation in aggressive behavior group into genetically correlated modules with shared functional annotations, sequence motifs, and tissue-specific expression. These modules are, in turn, correlated with other traits, providing insights about the molecular basis of pleiotropy between aggression and other behavioral and fitness-related traits. These results provide the foundation for a systems genetics analysis of natural variation in aggressive behavior. The future availability of whole genome DNA sequence variation for these lines will enable us to discriminate *cis*- from *trans*-acting polymorphisms, and infer the direction of the flow of information through the network. The entire suite of 266 candidate genes provides a focal point for linkage analysis of segregating populations derived from the inbred lines. Further, the inbred lines can be characterized for other quantitative traits, including components of metabolism, which will enable us to interpret the balance of selective forces maintaining variation for aggressive behavior in natural populations on a genome wide scale. Extension of these analyses to a larger sample of inbred lines will increase the power of network analyses, and provide a more representative sample of allelic diversity associated with aggressive behavior. Finally, it is not inconceivable that our understanding the genetic underpinnings of variation in aggressive behavior in *Drosophila *could be used to develop novel pharmacological therapies for treatment of pathological aggression in humans and domestic animals.

## Conclusions

Aggressive behavior is an important component of fitness in most animals, and is genetically complex, with natural variation attributable to multiple segregating loci with allelic effects that are sensitive to the physical and social environment. However, we know little about the genes and genetic networks affecting natural variation in aggressive behavior. We combined quantitative genetic analysis of variation in aggressive behavior with whole genome transcript profiling in a population of *D. melanogaster *inbred lines to identify 266 novel candidate genes associated with aggressive behavior, many of which have pleiotropic effects on metabolism, development, and/or other behavioral traits. Behavioral tests of mutations in 12 of these candidate genes showed that 9 indeed affected aggressive behavior. The genetically correlated transcripts formed a transcriptional genetic network of nine modules of correlated transcripts that are enriched for genes affecting common functions, tissue-specific expression patterns, and/or DNA sequence motifs. These results establish a foundation for understanding natural variation for complex behaviors in terms of networks of interacting genes.

## Materials and methods

### *Drosophila *strains

The 40 inbred lines were derived by 20 generations of full-sib mating from isofemale lines that were collected from the Raleigh, NC farmer's market in 2003 [21]. Flies were reared under standard culture conditions on cornmeal-molasses-agar medium at 25°C, 60 to 75% relative humidity, on a 12-h light-dark cycle. *P*-element insertional mutations and their co-isogenic control lines were obtained from Bloomington *Drosophila *Stock Center, Bloomington, Indiana, USA.

### Behavioral assay

Behavioral assays were performed as described previously [18] on socially experienced, 3- to 7-day-old males. Flies were not exposed to anesthesia for at least 24 h prior to the assay. A total of 20 replicate assays were performed for each line, with one replicate per line per day for a total of 20 days. Each replicate consisted of a group of eight 3- to 7-day-old flies of the same genotype. The flies were placed in a vial without food for 90 minutes, after which they were transferred (without anesthesia) to a test arena containing a droplet of food and allowed to acclimate for 2 minutes. After the acclimation period, the flies were observed for 2 minutes; the aggression score of each replicate was the total number of aggressive interactions observed among all eight flies in the 2-minute observation period. Behavioral assays were conducted in a behavioral chamber (25°C, 70% humidity) between 8 a.m. and 11 a.m.

### Whole genome expression analysis

The gene expression analysis has been described previously [21]. Briefly, RNA was extracted from two independent pools of 25 3- to 5-day-old mated whole flies/sex/line that were frozen at the same time of day, labeled, and hybridized to Affymetrix *Drosophila *2.0 arrays, using a strictly randomized experimental design. The raw array data were normalized using a median standardization. The measure of expression was the median log2 signal intensity of the probes in the perfect match probe sets, after removing probes containing SFPs between the wild-derived lines and the reference strain sequence used to design the array. Negative control probes were used to estimate the level of background intensity; probe sets with expression levels below this threshold were considered to be not expressed.

### Quantitative genetic analyses

The analysis of variance (ANOVA) model *Y *= *μ *+ *L *+ *ε *was used to partition variation in male aggressive behavior and transcript abundance between lines (*L*, random) and the variation within lines (*ε*). A FDR of < 0.01 [41] was used to assess significance of the *L *term in the analyses of natural variation in gene expression, to account for multiple testing. Broad sense heritabilities (*H*^2^) were estimated as:



- where *σ*_*L*_^2 ^and *σ*_*E*_^2 ^are the among line and within line variance components, respectively. Estimate of cross-trait genetic correlations were:



- where *cov*_*ij *_is the covariance of line means between trait *i *and trait *j*, and *σ*_*i *_and *σ*_*j *_are the square roots of the among line variance components for the two traits. Differences in aggressive behavior between *P*-element insert lines and their co-isogenic controls were assessed by *t*-tests, with significance levels based on Bonferroni-corrected *P*-values. Simple linear regressions were used to identify QTTs significantly associated (*P *< 0.01) with variation in aggressive behavior across the 40 lines. Similarly, ANOVA models (*Y *= *μ *+ *M *+ *ε*, where *M *denotes SFP presence or absence) were used to identify SFPs significantly associated (*P *< 0.05) with variation in aggressive behavior.

### Transcriptional networks

The genetic correlations between all transcripts significantly associated with aggressive behavior were computed after removing the correlation between these transcripts and the phenotype. This was achieved by fitting the model *Y *= *μ *+ *E *+ *ε*(*Y *is the phenotype and *E *is the covariate median log2 expression level) and extracting the residuals to compute the genetic correlations for module construction [21]. Modules of transcripts associated with aggressive behavior with coordinated patterns of expression across the 40 lines were then quantified as described previously [21] by transforming the pairwise genetic correlations among transcripts into Euclidean-like distances, which were used to construct an affinity matrix. The transcripts were partitioned into modules using a graph-theoretical approach that envisions the transcripts as nodes in an undirected graph whose edges are weighted by the entries of the affinity matrix. Transcriptional modules common to aggressive behavior and other phenotypes measured on the 40 wild-derived inbred lines [21,37] were identified by comparing the transcripts in each aggression module to the transcripts in each module from the other phenotypes, and determining whether the overlap between the modules exceed what is expected by chance using a Fisher's exact test [21].

### Bioinformatics

Statistical analyses were performed using JMP 7.0 software (SAS, Cary, NC, USA). Functional annotations of genes are based on FlyBase [38] annotations; additional information was obtained using FlyMine v12.0 [22] and Babelomics v2 and v3 [23]. Categories that were represented by fewer than 5% of the gene list queried were excluded. Statistically significant over- or under-representation was determined by the online software used when available; otherwise, a chi-square test was performed, using the appropriate genomic background to determine the expected values.

## Abbreviations

ANOVA: analysis of variance; FDR: false discovery rate; MEME: Multiple EM for Motif Elicitation; QTT: quantitative trait transcript; SFP: single feature polymorphism.

## Authors' contributions

TFCM and ACE designed research; ACE performed research; EAS contributed analytic tools; ACE and JFA analyzed data; TFCM and ACEF wrote the paper.

## Additional data files

The following additional data are available with the online version of this paper: transcripts significantly associated with variation in aggressive behavior among 40 wild-derived inbred lines (regression *P *< 0.01; Additional data file 1); associations of SFPs with aggressive behavior (Additional data file 2); Gene Ontologies represented by quantitative trait transcripts (Additional data file 3); Gene Ontologies represented by probe sets containing SFPs (Additional data file 4); Gene Ontology categories represented by genes associated with male aggressive behavior through either the identification of SFPs or transcript abundance (Additional data file 5); candidate genes previously associated with aggressive behavior [18] (Additional data file 6); analysis of modules of correlated transcripts associated with aggressive behavior (Additional data file 7); pleiotropic genes affecting aggression (Additional data file 8).

## Supplementary Material

Additional data file 1 Mean expression level, among (*σ*_*L*_^2^) and within (*σ*_*E*_^2^) line variance components, broad sense heritabilities (*H*^2^) and the false discovery rate (FDR) for the line term are for males only. Click here for file

Additional data file 2 The *P*-value is from the ANOVA of the difference in trait means between the two SFP classes. *a *is one half the difference in trait mean between the SFP alleles. MAF = minor allele frequency; MAS = mean aggression score. Click here for file

Additional data file 3 Level 3 **(a) **biological process and **(b) **molecular function Gene Ontology categories of genes for which variation in gene expression is correlated with variation in aggressive behavior. The percentage of genes falling into a given category is depicted on the x-axis. The relevant genomic background is the 7,508 probe sets that were differentially expressed in males at a FDR of < 0.01. No categories were significantly over-represented among level 3 categories; however, the level 4 'transport' biological process category was over-represented (adjusted *P *= 0.0364, data not shown). Click here for file

Additional data file 4 **(a) **Level 3 biological process and **(b) **level 4 molecular function categories. Only categories applying to at least 5% of a list are depicted. Categories significantly over-represented in the SFP list relative to the genomic background are denoted as follows: *adjusted *P *< 0.05; **P < 0.01; ***P < 0.001. Click here for file

Additional data file 5 Categories in **(a) **are level 3 biological processes; those in **(b) **are level 3 molecular functions. The percent of genes falling into a given category is depicted on the y-axis. Click here for file

Additional data file 6 Average |r| is the mean absolute value of the correlation of the transcript to all other variable transcripts. *H*^2 ^= broad sense heritability. QTT = quantitative trait transcript; SFP = single feature polymorphism. Click here for file

Additional data file 7 Degree = the average correlation of a transcript with all other transcripts in its module. Average degree = the average correlation of all transcripts in the module. Click here for file

Additional data file 8 Expression of these genes has been correlated with aggressive behavior and the other traits listed. **(a) **Reference [21]; (b) reference [37]. Click here for file

## References

[B1] Haller J, Kruk MR (2006). Normal and abnormal aggression: human disorders and novel laboratory models.. Neurosci Biobehav Rev.

[B2] Modesto-Lowe V, Brooks D, Ghani M (2006). Alcohol dependence and suicidal behavior: from research to clinical challenges.. Harv Rev Psychiatry.

[B3] Najt P, Perez J, Sanches M, Peluso MA, Glahn D, Soares JC (2007). Impulsivity and bipolar disorder.. Eur Neuropsychopharmacol.

[B4] Naudts K, Hodgins S (2006). Schizophrenia and violence: a search for neurobiological correlates.. Curr Opin Psychiatry.

[B5] Nelson RJ, Trainor BC (2007). Neural mechanisms of aggression.. Nat Rev Neurosci.

[B6] Kravitz EA, Huber R (2003). Aggression in invertebrates.. Curr Opin Neurobiol.

[B7] Young KA, Berry ML, Mahaffey CL, Saionz JR, Hawes NL, Chang B, Zheng QY, Smith RS, Bronson RT, Nelson RJ, Simpson EM (2002). *Fierce*: a new mouse deletion of *Nr2e1*; violent behaviour and ocular abnormalities are background-dependent.. Behav Brain Res.

[B8] Stork O, Welzl H, Cremer H, Schachner M (1997). Increased intermale aggression and neuroendocrine response in mice deficient for the *neural cell adhesion molecule *(*NCAM*).. Eur J Neurosci.

[B9] Alleva E, Cirulli F, Bianchi M, Bondiolotti GP, Chiarotti F, De Acetis L, Panerai AE (1998). Behavioural characterization of *interleukin-6 *overexpressing or deficient mice during agonistic encounters.. Eur J Neurosci.

[B10] Shigematsu N, Fukuda T, Yamamoto T, Nishioku T, Yamaguchi T, Himeno M, Nakayama KI, Tsukuba T, Kadowaki T, Okamoto K, Higuchi S, Yamamoto K (2008). Association of *cathepsin E *deficiency with the increased territorial aggressive response of mice.. J Neurochem.

[B11] Baier A, Wittek B, Brembs B (2002). *Drosophila *as a new model organism for the neurobiology of aggression?. J Exp Biol.

[B12] Jacobs ME (1978). Influence of beta-alanine on mating and territorialism in *Drosophila melanogaster*.. Behav Genet.

[B13] Chan YB, Kravitz EA (2007). Specific subgroups of FruM neurons control sexually dimorphic patterns of aggression in *Drosophila melanogaster*.. Proc Natl Acad Sci USA.

[B14] Vrontou E, Nilsen SP, Demir E, Kravitz EA, Dickson BJ (2006). *fruitless *regulates aggression and dominance in *Drosophila*.. Nat Neurosci.

[B15] Dierick HA, Greenspan RJ (2007). Serotonin and neuropeptide F have opposite modulatory effects on fly aggression.. Nat Genet.

[B16] Falconer DS, Mackay TF (1996). Introduction to Quantitative Genetics.

[B17] Dierick HA, Greenspan RJ (2006). Molecular analysis of flies selected for aggressive behavior.. Nat Genet.

[B18] Edwards AC, Rollmann SM, Morgan TJ, Mackay TF (2006). Quantitative genomics of aggressive behavior in *Drosophila melanogaster*.. PLoS Genet.

[B19] Passador-Gurgel G, Hsieh WP, Hunt P, Deighton N, Gibson G (2007). Quantitative trait transcripts for nicotine resistance in *Drosophila melanogaster*.. Nat Genet.

[B20] Winzeler EA, Richards DR, Conway AR, Goldstein AL, Kalman S, McCullough MJ, McCusker JH, Stevens DA, Wodicka L, Lockhart DJ, Davis RW (1998). Direct allelic variation scanning of the yeast genome.. Science.

[B21] Ayroles JF, Carbone MA, Stone EA, Jordan KW, Lyman RF, Magwire MM, Rollmann SM, Duncan LH, Lawrence F, Anholt RR, Mackay TF (2009). Systems genetics of complex traits in *Drosophila melanogaster*.. Nat Genet.

[B22] Lyne R, Smith R, Rutherford K, Wakeling M, Varley A, Guillier F, Janssens H, Ji W, McLaren P, North P, Rana D, Riley T, Sullivan J, Watkins X, Woodbridge M, Lilley K, Russell S, Ashburner M, Mizuguchi K, Micklem G (2007). FlyMine: an integrated database for *Drosophila *and *Anopheles *genomics.. Genome Biol.

[B23] Al-Shahrour F, Minguez P, Tarraga J, Montaner D, Alloza E, Vaquerizas JM, Conde L, Blaschke C, Vera J, Dopazo J (2006). BABELOMICS: a systems biology perspective in the functional annotation of genome-scale experiments.. Nucleic Acids Res.

[B24] Robin C, Daborn PJ, Hoffmann AA (2007). Fighting fly genes.. Trends Genet.

[B25] Dubnau J, Chiang AS, Grady L, Barditch J, Gossweiler S, McNeil J, Smith P, Buldoc F, Scott R, Certa U, Broger C, Tully T (2003). The staufen/pumilio pathway is involved in *Drosophila *long-term memory.. Curr Biol.

[B26] Yurkovic A, Wang O, Basu AC, Kravitz EA (2006). Learning and memory associated with aggression in *Drosophila melanogaster*.. Proc Natl Acad Sci USA.

[B27] Long AD, Lyman RF, Langley CH, Mackay TF (1998). Two sites in the *Delta *gene region contribute to naturally occurring variation in bristle number in *Drosophila melanogaster*.. Genetics.

[B28] Robin C, Lyman RF, Long AD, Langley CH, Mackay TF (2002). *hairy*: A quantitative trait locus for *Drosophila *sensory bristle number.. Genetics.

[B29] Jordan KW, Carbone MA, Yamamoto A, Morgan TJ, Mackay TF (2007). Quantitative genomics of locomotor behavior in *Drosophila melanogaster*.. Genome Biol.

[B30] Morozova TV, Anholt RR, Mackay TF (2007). Phenotypic and transcriptional response to selection for alcohol sensitivity in *Drosophila melanogaster*.. Genome Biol.

[B31] Toma DP, White KP, Hirsch J, Greenspan RJ (2002). Identification of genes involved in *Drosophila melanogaster *geotaxis, a complex behavioral trait.. Nat Genet.

[B32] Zemni R, Bienvenu T, Vinet MC, Sefiani A, Carrie A, Billuart P, McDonell N, Couvert P, Francis F, Chafey P, Fauchereau F, Friocourt G, des Portes V, Cardona A, Frints S, Meindl A, Brandau O, Ronce N, Moraine C, van Bokhoven H, Ropers HH, Sudbrak R, Kahn A, Fryns JP, Beldjord C, Chelly J (2000). A new gene involved in X-linked mental retardation identified by analysis of an X;2 balanced translocation.. Nat Genet.

[B33] Sheen VL, Ganesh VS, Topcu M, Sebire G, Bodell A, Hill RS, Grant PE, Shugart YY, Imitola J, Khoury SJ, Guerrini R, Walsh CA (2004). Mutations in *ARFGEF2 *implicate vesicle trafficking in neural progenitor proliferation and migration in the human cerebral cortex.. Nat Genet.

[B34] Sieberts SK, Schadt EE (2007). Moving toward a system genetics view of disease.. Mamm Genome.

[B35] Bailey TL, Williams N, Misleh C, Li WW (2006). MEME: discovering and analyzing DNA and protein sequence motifs.. Nucleic Acids Res.

[B36] Chintapalli VR, Wang J, Dow JA (2007). Using FlyAtlas to identify better *Drosophila melanogaster *models of human disease.. Nat Genet.

[B37] Harbison ST, Carbone MA, Ayroles JF, Stone EA, Lyman RF, Mackay TF (2009). Co-regulated networks that contribute to natural genetic variation in *Drosophila *sleep.. Nature Genetics.

[B38] Wilson RJ, Goodman JL, Strelets VB (2008). FlyBase: integration and improvements to query tools.. Nucleic Acids Res.

[B39] Hewes RS, Taghert PH (2001). Neuropeptides and neuropeptide receptors in the *Drosophila melanogaster *genome.. Genome Res.

[B40] Brody T, Cravchik A (2000). *Drosophila melanogaster *G protein-coupled receptors.. J Cell Biol.

[B41] Storey JD, Tibshirani R (2003). Statistical significance for genomewide studies.. Proc Natl Acad Sci USA.

